# The combination of herbal medicine Weng-li-tong with Tolterodine may be better than Tolterodine alone in the treatment of overactive bladder in women: a randomized placebo-controlled prospective trial

**DOI:** 10.1186/s12894-016-0167-1

**Published:** 2016-08-08

**Authors:** Dong-dong Xiao, Jian-wei Lv, Xin Xie, Xing-wei Jin, Mu-jun Lu, Yuan Shao

**Affiliations:** 1 Department of Urology, Shanghai Ninth People’s Hospital, Shanghai Jiao Tong University School of Medicine, Zhi-zao-ju Road, Shanghai, 200011 China; 2 Department of Urology, South Campus, Renji Hospital, School of Medicine, Shanghai Jiao Tong University, 2000 Jiang-yue Road, Shanghai, 201112 China; 3 Department of Urology, Ruijin Hospital North, School of Medicine, Shanghai Jiao Tong University, 999 Xi-wang Road, Shanghai, 201801 Peoples Republic of China; 4 Department of Urology and Andrology, Renji Hospital, School of Medicine, Shanghai Jiao Tong University, 145 Middle Shan-dong Road, Shanghai, 200001 Peoples Republic of China

**Keywords:** Overactive bladder, Herbal medicine, Weng-li-tong, Synergistic effect, Tolterodine

## Abstract

**Background:**

To assess the efficacy and safety of the herbal medicine, Weng-li-tong (WLT) as monotherapy or combined with tolterodine in women with overactive bladder (OAB).

**Methods:**

A prospective, randomized, single-blind multi-center trial was performed which included 182 OAB patients treated with either placebo (*n* = 26), WLT (*n* = 52), tolterodine (*n* = 52) or WLT plus tolterodine (*n* = 52). The overactive bladder symptom score (OABSS) and micturition behavior were measured to evaluate treatment efficacy.

**Results:**

In total, 146 patients [placebo (*n* = 23), WLT (*n* = 39), tolterodine (*n* = 41) and WLT plus tolterodine (*n* = 43)] completed 8 weeks of treatment. Compared to those treated with placebo, patients in three intervention groups showed significant improvements in the OABSS, voiding frequency, average voided volume and urgency incontinence. WLT had a slower onset than tolterodine or combination therapy in reducing urgency incontinence. Compared with tolterodine, WLT had a weaker effect in improving OABSS (*P* = 0.022) and daily voiding frequency (*P* = 0.034). The combination therapy had better efficacy than WLT or tolterodine alone in improving the OABSS, voiding frequency and voided volume. No significant differences in the changes in quality of life scores were observed among the three intervention groups. Residual urine increased significantly in tolterodine group (*P* = 0.004), but not in combination group. WLT resulted in fewer adverse effects than tolterodine such as dry mouth (*P* = 0.002), weak stream (*P* = 0.002) and less residual urine (*P* < 0.001).

**Conclusions:**

WLT could improve OAB symptoms in women, while it had slower onset and weaker efficacy but fewer adverse effects than tolterodine. The combination of WLT and tolterodine was more efficacious than tolterodine alone in improving OAB symptoms.

**Trial registration:**

Chinese Clinical Trial Registry [ChiCTR-IPR-14005626]. Date of registration: 7 December 2014.

## Background

Overactive bladder (OAB) is defined as a syndrome involving urinary urgency, usually accompanied by urinary frequency and nocturia, with or without urgency incontinence, in the absence of urinary tract infection or other obvious pathology [1].

Although behavioral modifications and pelvic floor physiotherapy are first-line options, pharmacologic therapy such as anticholinergic drugs is the mainstay of OAB treatment [2]. As OAB is a chronic disease requiring long-term treatment, adherence and compliance to drug usage is of great importance. Unfulfilled treatment expectation and poor adherence to anticholinergic medications remain a major challenge in the treatment of OAB [3] and lead to the use of over-the-counter alternative substances [4], of which herbal medication is one of the most preferred treatment alternatives or adjuvants to anticholinergic medications.

Weng-Li-Tong capsule (WLT, North China Pharmaceutical Group Corporation, Hebei, China; Sino-FDA Registration No. z19991104), a traditional Chinese medicine, is a mixture of herbal medicines, including Semen Coicis and Bulbus Fritillariae Thunbergii. In clinical practice, WLT can ameliorate the symptoms of urinary urgency and urinary frequency, and has anti-inflammatory effects in chronic prostatitis [5]. We designed the present clinical trial to determine if WLT could provide similar efficacy in women with OAB as an effective adjunctive therapy to anticholinergic medications. As tolterodine is a representative anticholinergic drug, it was included as the comparison of standard treatment.

## Methods

### Trial design

**Fig. 1:**
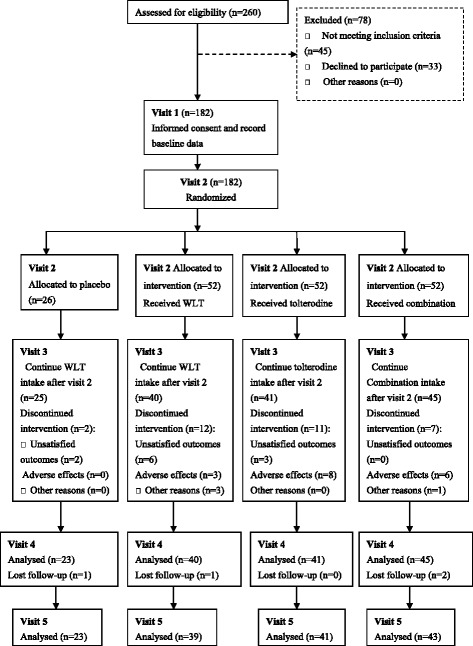
Protocol of patient enrolment and follow-up progress through this trial

This study was a randomized, single-blind (to patients) placebo-controlled, prospective multi-center trial, and was conducted at the Department of Urology of Shanghai Ninth People’s Hospital, Ruijin Hospital and Renji Hospital. Patient screening was started in January 2014. All participants were divided into the following four treatment groups: placebo (with same appearance of WLT), WLT alone, tolterodine alone and WLT combined with tolterodine at the ratio of 1:2:2:2 from December 2014 to June 2015 after the trial was finally approved by the ethics committee. The sample size was constructed to include 21 cases in the placebo group and 40 patients in each of the three intervention groups, aiming to detect a between-group difference in overactive bladder symptom score (OABSS) change of at least 10 % at 8 weeks with a two-sided significance level of 0.05 and a power of 0.9 (using the calculate tool website: http://powerandsamplesize.com/Calculators) (Fig. 1). The flow chart of this trial is shown in Fig. 1.

The trial was approved by the ethics committee of Shanghai Ninth People’s Hospital (Number: 201497) and registered at the Chinese Clinical Trial Registry (ChiCTR-IPR-14005626,http://www.chictr.org.cn/) and can be searched at WHO ICTRP Search Portal (http://apps.who.int/trialsearch/) using the registry number. Written informed consent was provided by each participant. In addition to the Handbook for Good Clinical Research Practice (GCRP) and the International Conference on Harmonization, this trial was also conducted in accordance with Shanghai local regulatory requirements and laws. Reporting of this trial was in accordance with the CONSORT 2010 checklist and CONSORT extension for herbal medicine interventions.

The primary outcome was reduction in the OABSS, and the secondary outcomes included a decrease in urgency incontinence and urinary frequency with improvement in quality of life.

### Inclusion criteria

The inclusion criteria for participants during the screening period were as follows: (1) female, aged from 18 to 75 years; (2) urinary urgency and urinary frequency, with or without urge incontinence; (3) average voiding volume < 200 ml; (4) urinalysis excluded urinary tract infection and represented no sign of urinary tract infection 4 weeks before screening; (5) patients were able to complete a micturition diary with good compliance and no previous anticholinergic usage. Exclusion criteria included: (1) severe cardiac diseases and arrhythmia; (2) severe hepatic and renal diseases, in which case aspartate aminotransferase, alanine aminotransferase and creatinine were 1.5 times greater than the upper limit of the normal reference range; (3) contraindications to anticholinergic therapy, such as urinary retention, gastric retention, uncontrolled angle-closure glaucoma or allergy to WLT; (4) acute urinary tract infection with symptoms during the screening period; (5) hematuria of unknown cause, cancer, interstitial cystitis, neurogenic bladder, bladder outlet obstruction, urethral catheterization, brain trauma, thyroid diseases, diabetes mellitus and other diseases which the researchers recognized as unsuitable for inclusion in this trial; (6) pregnancy, or preparation for pregnancy.

### Interventions

In total, 182 participants were recruited and 146 patients underwent five visits over 8 weeks, which included baseline screening at week 0 (visit 1), randomization, allocation and start of drug intake 3 days after baseline screening (visit 2) with follow up at week 2 (visit 3), week 4 (visit 4) and week 8 (visit 5) (Fig. 1).

The tolterodine group received extended-release tolterodine (4 mg per day, GSK, Nanjing, China), and the WLT group was treated with WLT (400 mg per capsule, three capsules twice daily). The combination group received WLT plus tolterodine at the same doses as described above.

### Components of the Weng-Li-Tong (WLT) capsule

One WLT capsule (400 mg) contains Ma-yuen Jobstears Seed (Semen Coicis, 66.3 mg), Chekiang Fritillary Bulb (Bulbus Fritillariae Thunbergii, 44.2 mg), Armand Clematis Stem (Caulis Clematidis Armandii, 39.8 mg), Cape Jasmine (Gardenia jasminoides Ellis, 39.8 mg), Japanese Honeysuckle Flower Bud (Flos Lonicerae, 44.2 mg), Japanese Inula Flower (Flos Inulae Japonicae, 39.8 mg), Hirsute Bugleweed Herb (Herba Lycopi Hirti, 39.8 mg), Verdigris (Mineralium Viridianum, 2.2 mg) and Liquorice Root (Radix Glycyrrhizae, 17.7 mg) with Radix Astragali (53 mg) and Radix Officinale (13.2 mg).

### Measurements

The patients’ perception of the impact of symptoms on their physical and emotional well-being is a major outcome parameter of OAB treatment. Measurement of patient-reported outcomes was aided by the OABSS [6], which included the frequency of micturition per day and per night, urinary urgency and urge incontinence. To determine bladder symptoms more precisely, a volume chart over three consecutive days was used in this research, and included data on micturition frequency (also included in OABSS), voided volume per micturition and episodes of urinary incontinence.

In addition to baseline clinical data, complete blood cell count, urinalysis, liver and renal panels, blood glucose and ultrasonography for residual urine were carried out at visit 1 and visit 5. The OABSS and the voiding volume chart were collected at start, 4 weeks and 8 weeks of treatment. Quality of life (QOL) scores were recorded at the begining and after 8 weeks of treatment using the scale of 0 (very well) to 5 (very bad) as a secondary endpoint.

### Safety assessments

Adverse events (AEs) were evaluated from 2 weeks to 8 weeks of treatment. The severity of each AE was determined by the Clavien grading system. A severe AE was considered unbearable and required withdrawal of the drug or medical intervention.

### Statistical analysis

All statistical analyses were performed using SPSS Statistics version 20.0 (SPSS Inc., Chicago, IL, USA). *P*-values were two-sided and considered statistically significant if less than 0.05. Baseline data in four groups were compared using the independent sample *t*-test for patients who completed the trial. After treatment,reductions in the OABSS, voids per 24 h and urinary incontinence with the changes in voided volume or residual urine were compared by ANOVA using the pairwise LSD test. The incidence of AEs was analyzed using the nonparametric Kruskal–Wallis test. Are these edits OK?

## Results

A total of 260 patients diagnosed with OAB were screened for eligibility. Of these, 182 patients who met the inclusion criteria were randomized and allocated into four groups with 26 patients in the placebo group and 52 patients in each of the three intervention groups. In total, 146 patients finished the treatment and follow-up periods: 23 in the placebo group, 39 in the WLT group, 41 in the tolterodine group and 43 in the combination group (Fig. 1). No significant differences in baseline demographic and clinical data, such as age, 24-h voiding frequency, average voided volume, urinary incontinence per 24 h and residual urine were observed between the groups (Table 1).

**Table 1:** Basic demographics and baseline clinical data

Group(*n*)	Placebo (26)	WLT (52)	Tolterodine (52)	Combination (52)	*P*-value
Age (y)	47(27–72)	49 (25–71)	48 (26–73)	48 (28–75)	0.832
OABSS	9.2 ± 2.6	8.9 ± 2.2	9.1 ± 2.8	9.6 ± 2.5	0.252
Voids/24 h	13.5 ± 2.9	13.0 ± 2.1	13.2 ± 2.8	13.8 ± 2.3	0.096
VV (ml)	140 ± 35	143 ± 22	141 ± 36	142 ± 31	0.887
Patients with UI	20	42	40	43	0.874
UI/24 h	1.10 ± 0.98	1.06 ± 0.96	0.97 ± 0.96	1.13 ± 1.05	0.783
RU (ml)	6.8 ± 9.7	5.6 ± 8.8	7.1 ± 9.8	6.2 ± 9.1	0.759

*WLT* weng-li-tong, *OABSS* overactive bladder symptom score, *VV* void volume, *UI* urinary incontinence, *RU* residual urine, *QOL* quality of life

### Efficacy

The patients in all three intervention groups showed significant improvement in the OABSS, frequency, average voided volume and urge incontinence after 8 weeks of treatment (Table 2).

**Table 2:** Efficacy comparison within groups between baseline, visit 4 (4 weeks) or visit 5 (8 weeks)

	Placebo^a^	WLT	*P*-value	Tolterodine	*P*-value	Combination	*P*-value
OABSS							
baseline	9.2 ± 2.6	8.9 ± 2.2		9.1 ± 2.8		9.6 ± 2.5	
visit 4	9.0 ± 2.8	8.0 ± 2.5	<0.001	8.0 ± 2.6	<0.001	7.4 ± 2.3	<0.001
visit 5	9.4 ± 2.7	8.2 ± 1.8	0.009	7.5 ± 2.7	<0.001	5.7 ± 2.0	<0.001
Voids/24 h							
baseline	13.5 ± 2.9	13.0 ± 2.1		13.2 ± 2.8		13.8 ± 2.3	
visit 4	13.2 ± 3.1	11.4 ± 2.0	<0.001	11.7 ± 2.6	<0.001	11.7 ± 2.3	<0.001
visit 5	13.3 ± 3.2	12.0 ± 2.3	0.012	11.0 ± 2.6	<0.001	10.0 ± 2.5	<0.001
VV (ml)							
baseline	140 ± 35	143 ± 22		141 ± 36		132 ± 42	
visit 4	135 ± 37	159 ± 33	<0.001	154 ± 38	<0.001	162 ± 35	<0.001
visit 5	136 ± 38	152 ± 24	0.025	167 ± 40	<0.001	192 ± 59	<0.001
Patients with UI							
baseline	18/23	31/39		32/41		35/43	
visit 4	16/23	29/39	0.788	24/41	0.096	27/43	0.091
visit 5	17/23	22/39	0.051	21/41	0.02	23/43	0.011
UI/24 h							
baseline	1.10 ± 0.98	1.06 ± 0.96		0.97 ± 0.96		1.13 ± 1.05	
visit 4	1.20 ± 1.08	1.04 ± 0.90	0.811	0.55 ± 0.70	<0.001	0.55 ± 0.60	<0.001
visit 5	1.18 ± 1.10	0.70 ± 0.65	<0.001	0.36 ± 0.51	<0.001	0.33 ± 0.40	<0.001
RU (ml)							
baseline	6.8 ± 9.7	5.6 ± 8.8		7.1 ± 9.8		6.2 ± 9.1	
visit 5	7.2 ± 10.2	4.9 ± 7.9	0.412	18.3 ± 29.7	0.004	6.4 ± 9.3	0.822

*WLT* weng-li-tong, *OABSS* overactive bladder symptom score, *VV* void volume, *UI* urinary incontinence, *RU* residual urine

^a^Placebo group showed no difference (all *P* > 0.05) of all parameters between baseline and visit 4/5

Combination therapy showed better efficacy than the other two intervention groups in terms of improvement in the OABSS, frequency and voided volume, while the reduction in urgency incontinence in the combination group was greater than that in the WLT group, but similar to that in the tolterodine group. Interestingly, the increase of residual urine was only significant in tolterodine group, but not in WLT or combination group (Table 3). An improvement in QOL is defined as a decrease in the QOL score of one or more. The number of intent-to-treat patients in the three intervention groups who improved at week 8 was 30 (57.9 %), 32 (61.5 %) and 40 (76.9 %), respectively, with no significant differences. However, the percentage of patients who improved in the intervention groups was much higher than that in the placebo group (3; 11.5 %) (all *P* <0.001) (Table 3).

**Table 3:** Efficacy comparison within groups at visit 5 (8 weeks)

Group (n)	Placebo^a^ (23)	WLT (39)	Tolterodine (41)	Combination (43)	*P*-value		
					W*vs* T	W *vs* C	T *vs* C
OABSS	0.22 ± 0.83	−0.72 ± 1.71	−1.54 ± 1.07	−3.9 ± 1.8	0.022	<0.001	<0.001
Voids/24 h	- 0.21 ± 0.77	−0.97 ± 2.3	−2.2 ± 1.6	−3.9 ± 2.1	0.034	<0.001	<0.001
VV (ml)	−4.8 ± 15.1	10.9 ± 24.4	24.6 ± 21.6	49.6 ± 43.9	0.135	<0.001	0.001
UI/24 h	0.02 ± 0.45	−0.36 ± 0.58	−0.61 ± 0.63	−0.78 ± 0.85	0.113	0.008	0.281
RU (ml)	0.5 ± 2.8	−0.9 ± 3.9	11.5 ± 23.2	0.2 ± 3.8	<0.001	0.109	<0.001
QOL improvement in ITT patients	11.5 %	57.9 %	61.5 %	76.9 %	0.842	0.059	0.136

*WLT* weng-li-tong, *OABSS* overactive bladder symptom score, *VV* void volume, *UI* urinary incontinence, *RU* residual urine, *QOL* quality of life, *ITT* intent-to-treat, *W* weng-li-tong, *T* tolterodine, *C* combination

^a^The changes of OABSS, voids/24 h, VV and UI with improve rate in placebo group were all significantly less than other 3 groups (all *P* < 0.001), except RU change was similar to WLT (*P* = 0.131) or combination group (*P* = 0.312)

Comparison between WLT and tolterodine showed that WLT had weaker efficacy than tolterodine in reducing the OABSS (*P* = 0.022) and voiding frequency per 24 h (*P* = 0.034). The percentage of patients with urgency incontinence decreased significantly in tolterodine or combination group, but marginally in WLT group (*P* = 0.051). WLT did not improve the episode of urgency incontinence at week 4 due to its slower onset, whereas it had a similar effect as tolterodine at week 8 (*P* = 0.113) (Table 3).

### Safety

The incidence of AEs in combination group and tolterodine group was similar, while AEs in WLT group were significantly fewer than those in tolterodine group which included dry mouth (*P* = 0.002), weak stream (*P* = 0.002) and less residual urine (*P* < 0.001). WLT resulted in a slightly higher incidence of diarrhea (12.8 %, *P* = 0.024) than tolterodine but was tolerable. No renal or hepatic malfunction or severe AEs were observed (Table 4).

**Table 4:** Adverse events comparison between intervention groups at visit 5 (8 weeks)

	Placebo (26)	Wenglitong (52)	Tolterodine (52)	Combination (52)	*P*		
					W vs T	W vs C	T vsC
Dry mouth	1	1	12	11	0.002	0.004	0.808
Weak stream	0	0	9	3	0.002	0.243	0.065
Diarrhea	0	5	0	3	0.024	0.468	0.241
Constipation	0	1	7	2	0.057	0.617	0.085
Stomach discomfort	2	4	3	7	0.709	0.525	0.314
Clavien system							
Grade 1	3	10	29	25	<0.001	0.003	0.229
Grade 2	0	1	2	1	0.586	0.944	0.529
Grade 3/4	0	0	0	0	--	--	--

*WLT* weng-li-tong, *W* weng-li-tong, *T* tolterodine, *C* combination

## Discussion

This is the first randomized clinical trial to investigate the efficacy and potential applicability of WLT as an adjunctive therapy to anticholinergic medication in the treatment of OAB in women. WLT improved OAB symptoms with better safety than tolterodine and showed a synergistic effect between WLT and tolterodine. The underlying pharmacological mechanism of WLT in the treatment of OAB should be investigated in the future, as this may indicate its mode of action.

According to a previous randomized, double-blind placebo-controlled trial, WLT was reported to have favorable therapeutic outcomes in patients with chronic prostatitis with acceptable side effects [7]. Originally targeting at benign prostate hyperplasia or prostatitis, WLT was reported to have analgesic and microcirculation-improving actions, and an anti-inflammatory effect in mouse models [8]. As aberrant neurogenic activity [9], cerebral alterations [10] and atypical or latent bladder infections [11] are involved in the pathogenesis of OAB, these factors may be controlled by WLT’s analgesic and anti-inflammatory effects. WLT could also block alpha1-receptor mediated vasoconstriction [12], thus reducing residual urine in combination with tolterodine.

The components of WLT may provide some insight into its pharmacological mechanism against OAB. Mineralium Viridianum was reported to have an important anti-inflammatory function [13] while Semen Coicis has an analgesic effect [14]. Peimine, extracted from Bulbus Fritillariae Thunbergii, inhibits the production of inflammatory cytokines induced by lipopolysaccharide (LPS) by blocking mitogen activated protein kinases (MAPKs) and NF-kB signaling pathways [15]. Caulis Clematidis Armandii has anti-neuroinflammatory activities [16]. Geniposide, extracted from Gardenia jasminoides Ellis, markedly inhibited LPS-induced TNF-α, IL-6 and IL-1β production both in vitro and in vivo [17]. Flos Lonicerae extracts exhibit antioxidant activity [18]. The anti-inflammatory activities of Flos Inulae Japonicae may be attributed to the inhibition of NO, iNOS and cytokine expression through the down-regulation of NF-kB activation via suppression of IkBα and MAPK phosphorylation in macrophages [19]. Luteolin-7-O-beta-D-glucuronide methyl ester was isolated and identified as the major compound in the ethyl acetate fraction of Herba Lycopi Hirti and had antioxidant activities [20]. The anti-inflammatory effect of Radix Glycyrrhizae is partially achieved by regulating the ERK signal pathway and inhibiting iNOS and COX-2 gene and protein expression through extracellular signals of MAPKs [21].

Some herbal medicines have been reported to have potential therapeutic effects in the treatment of OAB or lower urinary symptoms [22, 23]. However, there are few clinical trials of traditional Chinese medicines in the treatment of OAB. Our data indicated that WLT markedly reduced OABSS as the primary endpoint. For secondary endpoints of this trial, it could also decrease urinary frequency or urgency incontinence with voiding volume increase. Moreover, WLT improved QOL in 57.9 % of intent-to-treat patients. Despite its slower onset and weaker efficacy compared to tolterodine, WLT had fewer side effects. A synergistic effect between WLT and tolterodine could enhance the benefit of antimuscarinics in long-term therapy and prevent an increase in residual urine. WLT could probably be an alternative agent in OAB women who are unsuitable for anticholinergic drugs.

The limitations of this study include the small number of patients and short duration of treatment. A large prospective double-blind randomized placebo-controlled study is needed to confirm the efficacy of WLT in the clinical management of OAB.

## Conclusions

WLT could improve OAB symptoms in women, while it had slower and weaker efficacy but fewer adverse effects than tolterodine. The combination of WLT and tolterodine was more efficacious than tolterodine alone in improving OAB symptoms, indicating possible synergy between WLT and anticholinergic agents.

## Abbreviations

AEs, adverse events; GCRP, good clinical research practice; LPS, lipopolysaccharide; MAPKs, mitogen activated protein kinases; OAB, overactive bladder; OABSS, overactive bladder symptom score; QOL, quality of life; WLT, weng-li-tong
